# Association of body mass index and waist circumference with high blood pressure in older adults

**DOI:** 10.1186/s12877-021-02154-5

**Published:** 2021-04-19

**Authors:** Wenli Zhang, Kun He, Hao Zhao, Xueqi Hu, Chunyu Yin, Xiaoyan Zhao, Songhe Shi

**Affiliations:** 1 Department of Epidemiology and Health Statistics, College of Public Health, Zhengzhou University, Songhe Shi, 100 Kexue Avenue, Zhengzhou, Henan 450001 People’s Republic of China; 2 Department of Neurology, Chinese People’s Liberation Army General Hospital, Beijing, People’s Republic of China

**Keywords:** High blood pressure, Body mass index, Waist circumference, Restricted cubic spline, Additive interaction

## Abstract

**Background:**

The relationship between obesity and prevalent high blood pressure in older adults has predominantly been estimated using categorical measures of body mass index (BMI) and waist circumference (WC), masking the shape of the dose-response relationship. We aimed to examine the precise relationship of BMI, WC with high blood pressure and to assess the appropriate level of BMI and WC for high blood pressure.

**Methods:**

We examined data for 126,123 individuals in Xinzheng city aged ≥60 years from a population based study from January to December 2019. Logistic regression and restricted cubic spline models were applied to assess the relationship and the appropriate level of BMI and WC for high blood pressure. An additive interaction analysis was used to test synergistic effects between a higher BMI and WC for high blood pressure.

**Results:**

The full-adjusted odds ratios (ORs) with 95% confidence intervals (CIs) of an increase of 1 kg/m^2^ in BMI and 1 cm in WC for high blood pressure were 1.084 (1.080–1.087) and 1.026(1.024–1.027), respectively. Multivariable adjusted restricted cubic spline analyses showed the nonlinear relationships of BMI and WC with high blood pressure in both men and women (all *P* < 0.001). The risk of high blood pressure increased steeply with increasing BMI from ≥25 kg/m^2^ and WC ≥ 88 cm or 86 cm for males and females, respectively. And we observed a significant additive interaction between a higher BMI and WC such that the prevalence of high blood pressure was significantly enhanced.

**Conclusion:**

These findings suggest increased high blood pressure prevalence in the older adults with increased BMI and WC. BMI ≤ 25 kg/m2 and WC ≤ 88 cm or 86 cm for males and females may be the best suggestion with regard to primary prevention of high blood pressure in older adults.

**Supplementary Information:**

The online version contains supplementary material available at 10.1186/s12877-021-02154-5.

## Background

Hypertension is a substantial public health burden [1] and a strong modifiable risk factor for cardiovascular disease [2], which has become one of the leading causes of global mortality, accounting for 9.4 million deaths each year [3, 4]. From 2010 to 2017, the weighted prevalence of hypertension increased by 23.4% and is increasing with aging in China [4]. Obesity is one of the modifiable risk factors associated with cardiovascular disease [5–7], which is most generally assessed by body mass index (BMI) and waist circumference (WC). Commonly, BMI is used as a substitute for general obesity because of its strong correlation with gold standard body fat [8–10], and WC is regarded as a better index for evaluating abdominal obesity [11].

Accumulating evidence suggests a potential link between obesity-related high blood pressure (HBP) [12–14], but controversy exists about the degree of the associations between two anthropometric indicators and the risk of HBP. And information about the relationship between BMI, WC and HBP is limited in older adults. In particular, few studies have explored the dose–response relationship and investigated the interaction between BMI and WC. In addition, higher BMI and WC increase the risk of HBP alone, but their interrelations with an increased risk of HBP remain uncertain.

Thus, in this study, we aimed to assess the independent association of BMI and WC with HBP as well as their possible additive interactions on the risk of HBP using a large and contemporary population in central China and to explore the appropriate level of BMI and WC for the older adults.

## Methods

### Study population

**Fig. 1:**
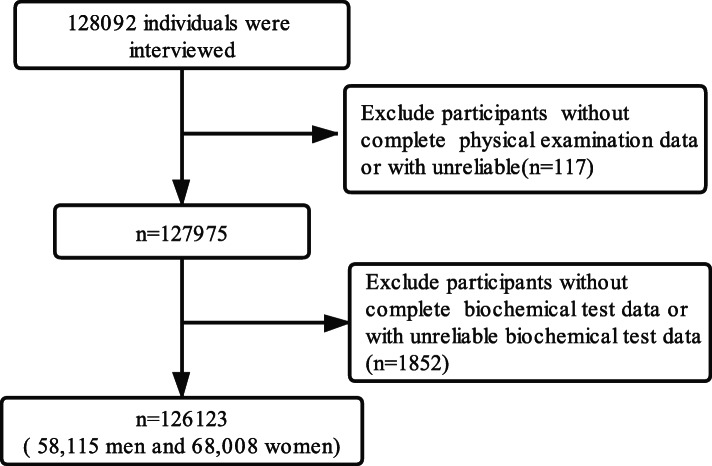
Flow diagram of the selection of eligible participants

The study population consisted of participants of a comprehensive health check-up program conducted at fourteen medical examination centers (Supplementary 1). Generally, the Central People’s Government of China demands that residents aged ≥60 years participate in health examinations annually to promote good health by enabling early detection of chronic diseases and associated risk factors. The study collected clinical, demographic, and lifestyle information from all participants by face-to-face interviews, physical examinations and blood biochemical examinations. Cross-sectional study data from Xinzheng from January to December 2019 were combined for analyses. For this study, 1969 people were excluded from the current study due to missing physical examination data (*n* = 117) or biochemical test data (*n* = 1852). Finally, we had data for 58,115 men and 68,008 women resident ≥60 years of age, who were enrolled to assess the association between BMI, WC and HBP (Fig. 1). Written informed consent was obtained from each participant before data collection. The research ethics committee of Zheng Zhou University approved the current study methodology, protocol, and procedures. (Reference Number: ZZUIRB2019–019).

### Exposure

Height, weight, and WC were measured twice by trained nurses following rigorous protocols. Body height was measured without shoes with a stadiometer, and body weight was measured with participants in light clothing and without shoes by electronic scales. WC was measured with gentle breathing at the midpoint between the lowest rib and the iliac crest to the nearest 0.1 cm. BMI was calculated as weight (kg) divided by the square of height (m). BMI was categorized into quintiles (Male: < 22.20, 22.20 ~ 24.02, 24.02 ~ 25.73, 25.73 ~ 27.78, ≥27.78 Female: < 22.43, 22.43 ~ 24.42, 24.4 ~ 26.27, 26.27 ~ 28.58, ≥28.58). And WC was categorized into quintiles (Male:< 81,81 ~ 86,86 ~ 90,90 ~ 96,≥96Female:< 79,79 ~ 84,84 ~ 89,89 ~ 95,≥95).

### Outcome

Systolic blood pressure (SBP) and diastolic blood pressure (DBP) were collected according to the World Health Organization definition [15]. After sitting quietly for five minutes, certified nurses measured each participant’s seated blood pressure three times using a mercury sphygmomanometer (Omron HEM-7125, Kyoto, Japan). We calculated the means of SBP and DBP. Diagnosis of HBP was defined as SBP ≥ 140 mmHg/or DBP ≥90 mmHg or use of antihypertensive medication within 2 weeks [15].

### Covariates

Information on demographic characteristics (age, sex, marital status, and place of residence) and behavioral measures (smoking, physical exercise and alcohol consumption) were obtained by a standardized questionnaire, which was in strict accordance with the *National Standards for Basic Public Health Services (2011)*. Marital status was categorized as unmarried, married, divorced and death of a spouse. Smoking status was defined as current smoker, former smokers, or never smoker [16]; Alcohol consumption and physical exercise status were categorized as never, once in a while, more than once a week and every day. Place of residence (rural and urban) was defined according to country-specific definitions. Stroke was defined as sudden onset of a focal, non-convulsive neurological deficit persisting longer than 24 h. Diagnosis of psychotic illnesses was met Diagnostic and Statistical Manual of Mental Disorders (DSM − 5) criteria for a diagnosis within the spectrum of primary psychotic illnesses. Diagnosis of cancer based on the International Classification of Diseases. Overnight fasting blood samples were collected into vacuum tubes for assessing serum levels of blood glucose using standard methods.

### Statistical analysis

Continuous data were expressed as the mean ± standard deviation (SD) and categorical data as the number (percentage) for quintiles of BMI or WC, using different values for men and women. Comparisons of the basic characteristics of the quintiles were performed with the *χ*^*2*^test, ANOVA, and Kruskal-Wallis test.

Logistic regression models were used to estimate the adjusted odds ratios (ORs) with 95% confidence intervals (CIs) for BMI, WC and HBP, taking the quintile with the lowest baseline BMI or WC values as the reference. ORs and 95% CIs for HBP were estimated for each group with adjustment of multiple confounders. One obesity parameter was introduced at a time in each model to avoid the collinear effect. Model 1 was unadjusted. Model 2 was adjusted for sex and age. Model 3 was adjusted for Model 2 and marital status, alcohol consumption, smoking, physical activity, place of residence, cancer, stroke, psychotic illness and blood glucose. A linear trend test was performed by modeling the median value of each exposure category as a continuous variable in the models. Fully adjusted restricted cubic spline analyses were used to characterize the dose-response association and explore the potential linear or nolinear relationship of BMI, and WC with HBP. The knots were placed at the 5th, 25th, 50th, 75th and 95th percentiles. The test result for overall association was checked first. If the test for overall association was significant, the test result for nonlinearity and linearity were checked, and the *P*-value for non-linear association < 0.05 indicated a significant result indicating the linear association. We also evaluated the additive interaction between BMI and WC for HBP with BMI and WC analyzed as continuous variable in two categories [(BMI: BMI < 25 and BMI ≥ 25. WC: WC < 102 for males, WC < 88 for females and WC ≥ 102 for males, WC ≥ 88 for females) or (WHO proposed cut-off points [17])]. We applied three indicators to evaluate the additive interaction: relative excess risk due to interaction (RERI), attributable proportion due to interaction (AP) or synergy index (S). RERI > 0, AP > 0, or S > 1 was regarded as a significant additive interaction.

Statistical analyses involved the use of SAS V .9.1 (SAS Institute) and R × 64 4.0.0.All reported *P* values were two-sided, with *P* < 0.05 considered statistically significant.

## Results

### Basic demographic characteristics

A total of 126,123 participants were eligible for inclusion in this study. The mean (SD) age was 70.29 (6.94) years. The basic demographic characteristics of the study population according to BMI and WC quintiles are shown in Tables 1 and 2. The prevalence of HBP and stroke, levels of alcohol consumption, physical exercise and blood glucose showed a significant progressive increase from the quintile with the lowest BMI to the quintile with the highest BMI. This was also the case for the WC quintiles.

**Table 1:** Basic demographic characteristics of Subjects According to BMI Quintiles

Characteristics	First Quintile	Second Quintile	Third Quintile	Fourth Quintile	Fifth Quintile	*P* for trend
Male	BMI < 22.20	22.20 ≤ BMI < 24.02	24.02 ≤ BMI < 25.73	25.73 ≤ BMI < 27.78	BMI ≥ 27.78	
Female	BMI < 22.43	22.43 ≤ BMI < 24.42	24.42 ≤ BMI < 26.27	26.27 ≤ BMI < 28.58	BMI ≥ 28.58	
N0.	25,164	25,236	25,166	25,268	25,289	
High blood pressure						< 0.001
no	13,812 (54.89)	12,206 (48.37)	11,346 (45.08)	10,508 (41.59)	9205 (36.4)	
yes	11,352 (45.11)	13,030 (51.63)	13,820 (54.92)	14,760 (58.41)	16,084 (63.6)	
Cancer						0.005
no	25,137 (99.89)	25,215 (99.92)	25,146 (99.92)	25,246 (99.91)	25,282 (99.97)	
yes	27 (0.11)	21 (0.08)	20 (0.08)	22 (0.09)	7 (0.03)	
Stroke						0.003
no	25,036 (99.49)	25,099 (99.46)	25,056 (99.56)	25,163 (99.58)	25,194 (99.62)	
yes	128 (0.51)	137 (0.54)	110 (0.44)	105 (0.42)	95 (0.38)	
Psychotic illness						0.009
no	24,982 (99.28)	25,107 (99.49)	25,027 (99.45)	25,177 (99.64)	25,136 (99.39)	
yes	182 (0.72)	129 (0.51)	139 (0.55)	91 (0.36)	153 (0.61)	
Age, years	72.08 ± 7.75	70.54 ± 7.17	69.96 ± 6.66	69.59 ± 6.49	69.29 ± 6.17	< 0.001
sex,%						0.981
men	11,619 (46.17)	11,584 (45.9)	11,612 (46.14)	11,642 (46.07)	11,658 (46.1)	
women	13,545 (53.83)	13,652 (54.1)	13,554 (53.86)	13,626 (53.93)	13,631 (53.9)	
Marital status, %						< 0.001
unmarried	558 (2.22)	432 (1.71)	341 (1.36)	285 (1.13)	246 (0.97)	
married	19,847 (78.87)	20,968 (83.09)	21,315 (84.7)	21,686 (85.82)	21,916 (86.66)	
divorced	135 (0.54)	91 (0.36)	124 (0.49)	92 (0.36)	83 (0.33)	
death of a spouse	4624 (18.38)	3745 (14.84)	3386 (13.45)	3205 (12.68)	3044 (12.04)	
Smoking, %						< 0.001
Never smokers	20,950 (83.25)	21,506 (85.22)	21,557 (85.66)	21,879 (86.59)	22,079 (87.31)	
Former smokers	537 (2.13)	591 (2.34)	611 (2.43)	623 (2.47)	678 (2.68)	
Current smokers	3677 (14.61)	3139 (12.44)	2998 (11.91)	2766 (10.95)	2532 (10.01)	
Alcohol consumption, %						< 0.001
Never	23,681 (94.11)	23,651 (93.72)	23,364 (92.84)	23,379 (92.52)	23,296 (92.12)	
Once in a while	906 (3.6)	998 (3.95)	1113 (4.42)	1129 (4.47)	1215 (4.8)	
More than once a week	243 (0.97)	277 (1.1)	305 (1.21)	350 (1.39)	352 (1.39)	
Every day	334 (1.33)	310 (1.23)	384 (1.53)	410 (1.62)	426 (1.68)	
Physical exercise, %						< 0.001
Never	16,555 (65.79)	15,881 (62.93)	15,014 (59.66)	14,897 (58.96)	15,290 (60.46)	
Once in a while	863 (3.43)	912 (3.61)	943 (3.75)	998 (3.95)	966 (3.82)	
More than once a week	1576 (6.26)	1809 (7.17)	1937 (7.7)	1914 (7.57)	1830 (7.24)	
Every day	6170 (24.52)	6634 (26.29)	7272 (28.9)	7459 (29.52)	7203 (28.48)	
Residence						< 0.001
Rural areas	21,838 (86.78)	20,703 (82.04)	20,322 (80.75)	20,314 (80.39)	20,365 (80.53)	
Urban areas	3326 (13.22)	4533 (17.96)	4844 (19.25)	4954 (19.61)	4924 (19.47)	
Blood glucose, mmol/L	5.44 ± 1.8	5.68 ± 1.86	5.82 ± 1.91	5.91 ± 1.91	6.04 ± 1.95	< 0.001

Data are shown as mean ± SD or n (%). *BMI* body mass index, Residence, area of residence; Blood glucose, Fasting plasma glucose

**Table 2:** Basic demographic characteristics of Subjects According to WC Quintiles

Characteristics	First Quintile	Second Quintile	Third Quintile	Fourth Quintile	Fifth Quintile	*P* for trend
Male	WC < 81	81 ≤ WC < 86	86 ≤ WC < 90	90 ≤ WC < 96	WC ≥ 96	
Female	WC < 79	79 ≤ WC < 84	84 ≤ WC < 89	89 ≤ WC < 95	WC ≥ 95	
N0.	23,098	23,498	24,971	26,736	27,820	
High blood pressure						< 0.001
no	12,346 (53.45)	11,421 (48.6)	11,219 (44.93)	11,539 (43.16)	10,552 (37.93)	
yes	10,752 (46.55)	12,077 (51.4)	13,752 (55.07)	15,197 (56.84)	17,268 (62.07)	
Age, years	71.29 ± 7.65	70.48 ± 7.15	69.96 ± 6.79	69.86 ± 6.57	70.01 ± 6.51	< 0.001
sex,%						0.001
men	10,850 (46.97)	10,887 (46.33)	10,190 (40.81)	13,076 (48.91)	13,112 (47.13)	
women	12,248 (53.03)	12,611 (53.67)	14,781 (59.19)	13,660 (51.09)	14,708 (52.87)	
Cancer						0.076
no	23,075 (99.9)	23,479 (99.92)	24,949 (99.91)	26,720 (99.94)	27,803 (99.94)	
yes	23 (0.1)	19 (0.08)	22 (0.09)	16 (0.06)	17 (0.06)	
Stroke						0.008
no	22,974 (99.46)	23,375 (99.48)	24,869 (99.59)	26,623 (99.58)	27,707 (99.59)	
yes	124 (0.54)	123 (0.52)	102 (0.41)	113 (0.42)	113 (0.41)	
Psychotic illness						0.091
no	22,937 (99.3)	23,384 (99.51)	24,835 (99.46)	26,613 (99.54)	27,660 (99.42)	
yes	161 (0.7)	114 (0.49)	136 (0.54)	123 (0.46)	160 (0.58)	
Marital status, %						< 0.001
unmarried	546 (2.36)	427 (1.82)	312 (1.25)	289 (1.08)	288 (1.04)	
married	18,446 (79.86)	19,404 (82.58)	21,106 (84.52)	22,908 (85.68)	23,868 (85.79)	
divorced	120 (0.52)	99 (0.42)	106 (0.42)	103 (0.39)	97 (0.35)	
death of a spouse	3986 (17.26)	3568 (15.18)	3447 (13.8)	3436 (12.85)	3567 (12.82)	
Smoking, %						< 0.001
Never smokers	19,441 (84.17)	19,920 (84.77)	21,755 (87.12)	22,749 (85.09)	24,106 (86.65)	
Former smokers	469 (2.03)	534 (2.27)	555 (2.22)	748 (2.8)	734 (2.64)	
Current smokers	3188 (13.8)	3044 (12.95)	2661 (10.66)	3239 (12.11)	2980 (10.71)	
Alcohol consumption, %						< 0.001
Never	21,794 (94.35)	22,082 (93.97)	23,500 (94.11)	24,555 (91.84)	25,440 (91.45)	
Once in a while	799 (3.46)	913 (3.89)	927 (3.71)	1322 (4.94)	1400 (5.03)	
More than once a week	208 (0.9)	217 (0.92)	233 (0.93)	406 (1.52)	463 (1.66)	
Every day	297 (1.29)	286 (1.22)	311 (1.25)	453 (1.69)	517 (1.86)	
Physical exercise, %						< 0.001
Never	14,960 (64.77)	14,768 (62.85)	15,405 (61.69)	15,830 (59.21)	16,674 (59.94)	
Once in a while	808 (3.5)	835 (3.55)	996 (3.99)	998 (3.73)	1045 (3.76)	
More than once a week	1507 (6.52)	1790 (7.62)	1871 (7.49)	1962 (7.34)	1936 (6.96)	
Every day	5823 (25.21)	6105 (25.98)	6699 (26.83)	7946 (29.72)	8165 (29.35)	
Residence						< 0.001
Rural areas	20,201 (87.46)	20,096 (85.52)	20,773 (83.19)	21,110 (78.96)	21,362 (76.79)	
Urban areas	2897 (12.54)	3402 (14.48)	4198 (16.81)	5626 (21.04)	6458 (23.21)	
Blood glucose, mmol/L	5.44 ± 1.71	5.64 ± 1.81	5.76 ± 1.88	5.9 ± 1.97	6.08 ± 2.01	< 0.001

Data are shown as mean ± SD or n (%). *WC* waist circumference; Residence, area of residence; Blood glucose, Fasting plasma glucose

### OR and 95% confidence intervals (CIs) for HBP according to BMI and WC

Table 3 presents the results from the logistic regression that estimated the association between the levels of BMI, WC and HBP. The multivariable adjusted OR (95% CI) per 1 kg/m^2^ increase in BMI was 1.084 (1.08 to 1.087). In all three models, the ORs for HBP increased significantly with increasing BMI quintiles (*P* for trend < 0.01). In Model 3, the multivariate-adjusted OR (95% CIs) for HBP with the highest BMI quintile group compared with the lowest quintile group was 2.300(2.217 to 2.386). The multivariable adjusted OR (95% CI) per 1 cm increase in WC was 1.025 (1.024 to 1.027). The highest quintile group had a greater HBP prevalence than the other quintile group, and the crude ORs (95% CIs) for HBP compared with the lowest quintile group was 1.879 (1.814 to 1.947). After adjustment for sex, age, marital status, alcohol consumption, smoking, physical activity, place of residence and blood glucose, the ORs were enhanced, and the multivariate-adjusted ORs (95% CIs) for HBP with the highest WC quintile group compared with the lowest quintile group was 1.977(1.906 to 2.050).

**Table 3:** Association between BMI, WC and HBP

Characteristics	Model 1	Model 2	Model 3
	OR(95%CI)	OR(95%CI)	OR(95%CI)
BMI,per1kg/m^2^ change	1.077 (1.074,1.08)	1.085 (1.081,1.088)	1.084 (1.080,1.087)
BMI group			
Q1	1.000(ref)	1.000(ref)	1.000(ref)
Q2	1.299 (1.254,1.345)	1.361 (1.313,1.41)	1.363 (1.316,1.413)
Q3	1.482 (1.431,1.535)	1.581 (1.525,1.638)	1.580 (1.524,1.637)
Q4	1.709 (1.65,1.77)	1.844 (1.779,1.911)	1.835 (1.770,1.903)
Q5	2.126 (2.051,2.203)	2.317 (2.235,2.403)	2.300 (2.217,2.386)
*P* for trend	< 0.001	< 0.001	< 0.001
WC,per1 cm change	1.022 (1.021,1.024)	1.025 (1.024,1.026)	1.026 (1.024,1.027)
WC group			
Q1	1.000(ref)	1.000(ref)	1.000(ref)
Q2	1.214 (1.171,1.259)	1.239 (1.195,1.286)	1.237 (1.192,1.283)
Q3	1.408 (1.358,1.459)	1.443 (1.391,1.496)	1.445 (1.394,1.499)
Q4	1.512 (1.46,1.567)	1.577 (1.522,1.635)	1.593 (1.537,1.652)
Q5	1.879 (1.814,1.947)	1.95 (1.881,2.021)	1.977 (1.906,2.050)
*P* for trend	< 0.001	< 0.001	< 0.001

*BMI* body mass index, *WC* waist circumference. Model1: unadjusted. Model2: adjusted for sex and age. Model3 adjusted for Model2 and marital status, alcohol consumption, smoking, physical activity, place of residence,cancer, stroke, psychotic illness and blood glucose. Linear trend test was performed by modeling the median value of each exposure category as a continuous variable in the models

### Dose response analysis

**Fig. 2:**
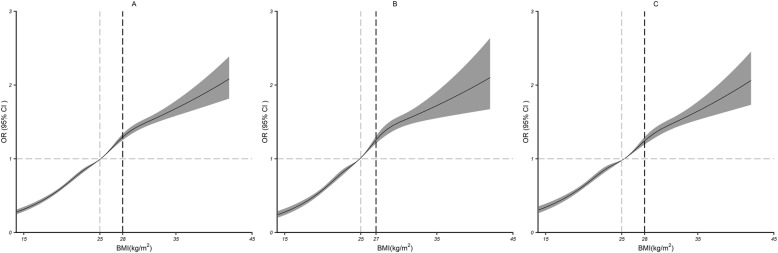
Dose–response relationship between BMI and HBP. BMI and HBP outcomes in the total study population (**a**), male study population (**b**) and female study population (**c**). The associations were adjusted for age, marital status, alcohol consumption, smoking, physical activity, area, cancer, stroke, psychotic illness and blood glucose. The solid lines and gray areas represent the estimated ORs and their 95% CIs. The gray dashed lines represent the corresponding BMI when the OR is 1, and the black dashed lines represent the point where the slope suddenly decreases the most, corresponding to the BMI

Multivariable adjusted restricted cubic spline analyses showed the nonlinear relationships of BMI with HBP (all *P* < 0.001; Fig. 2a). The risk of HBP increased with increasing BMI. As BMI increased, the ORs increased from 0.31(0.28 to 0.35) to 2.08(1.82 to 2.39) in the 15–42 kg/m^2^ range. As a result, the ORs were inversely associated with HBP when BMI was below 25 kg/m^2^, but presented a significant risk effect above this value. Subgroup analyses on men-women did not show significant differences (Fig. 2b; c).

**Fig. 3:**
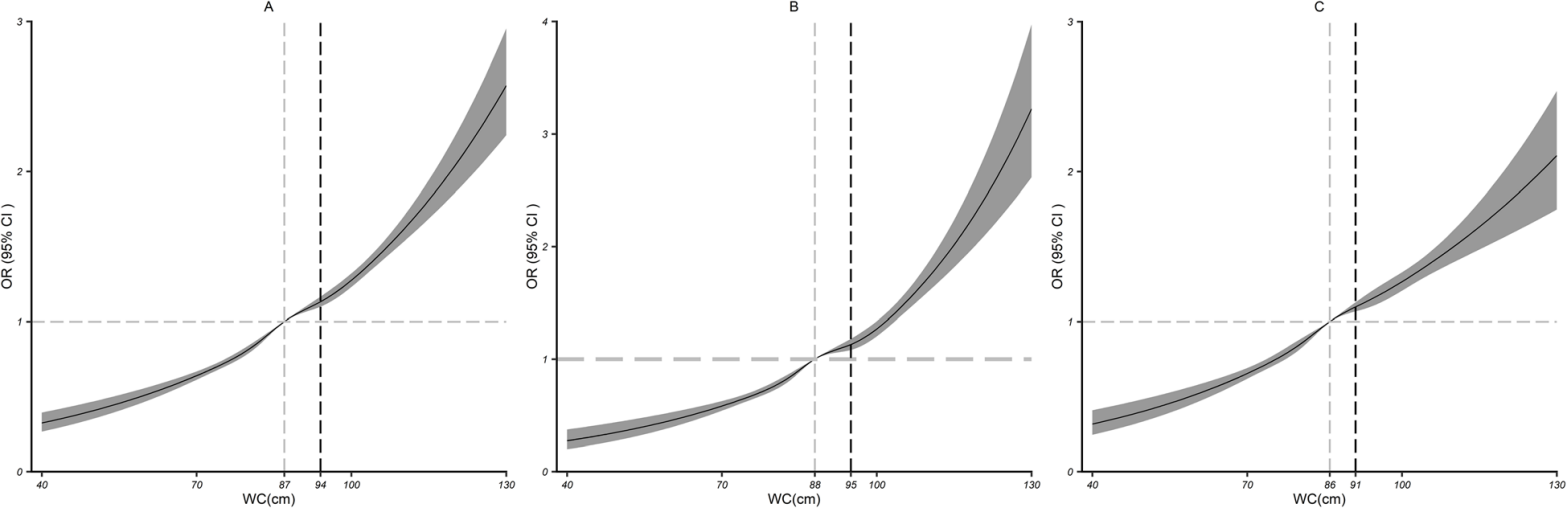
Dose–response relationship between WC and HBP. WC and HBP outcomes in the total study population (**a**), male study population (**b**) and female study population (**c**). The associations were adjusted for age, marital status, alcohol consumption, smoking, physical activity, area, cancer, stroke, psychotic illness and blood glucose (serum levels of glucose). The solid lines and gray areas represent the estimated ORs and their 95% CIs. The gray dashed lines represent the corresponding WC when the OR is 1, and the black dashed lines represent the WC corresponding to the point where the slope changes the most

The results of the dose-response relationship analysis between WC and HBP are shown in Fig. 3a. A nonlinear association (all *P* < 0.001) between WC and HBP was detected. As WC increased, the ORs increased from 0.32(0.27 to 0.39) to 2.57(2.24 to 2.95) in the 40–130 cm range. When stratified by sex, the ORs increased from 0.27(0.20 to 0.37) to 3.22(2.62 to 3.97) in the male population. With an increase in WC, when WC was over 88 cm and 86 cm for males and females, respectively, WC was more steeply positively associated with the risk of HBP (Fig. 3b; c).

### Additive interaction analysis

Table 4 presents the results from additive interaction analysis. We observed a significant additive interaction between higher BMI and WC such that the prevalence of HBP increased (RERI = 1.28, 95% CI: 1.13–1.43; AP = 0.43, 95% CI: 0.41–0.45; S = 2.88, 95% CI: 2.79–2.97). If BMI < 25.0 kg/m^2^ and WC < 88 cm for males and WC < 86 cm for females were used as the reference, BMI ≥ 25 alone and WC ≥ 88 for males or WC ≥ 86 for females alone were both associated with increased risks of HBP. The copresence of both factors greatly enhanced the adjusted ORs of higher BMI alone 1.476(1.418 to 1.536) and higher WC alone 1.230(1.186 to 1.275) to 1.729(1.685 to 1.775) for HBP, with significant additive interactions. When BMI and WC were classified by the proposed cut-off points [17] on the waist circumference continuum (BMI: 25 kg/m^2^, WC: 88 cm for females and 102 cm for males), there was still significant additive interaction (RERI = 1.68, 95% CI: 1.48–1.87; AP = 0.48, 95% CI: 0.46–0.50; S = 3.00, 95% CI: 2.91–3.10).

**Table 4:** Additive interaction analysis of BMI and WC for HBP

Characteristics	Model 1	Model 2	Model 3
	OR(95%CI)	OR(95%CI)	OR(95%CI)
Additive interaction models1			
BMI < 25 and WC < 88/102	1.000(ref)	1.000(ref)	1.000(ref)
BMI ≥ 25 and WC < 88/102	1.459 (1.42,1.5)	1.546 (1.503,1.589)	1.517 (1.476,1.56)
BMI < 25 and WC ≥ 88/102	1.377 (1.304,1.453)	1.286 (1.216,1.36)	1.301 (1.232,1.375)
BMI ≥ 25 and WC ≥ 88/102	1.749 (1.699,1.799)	1.757 (1.705,1.811)	1.771 (1.719,1.823)
Additive interaction models2			
BMI < 25 and WC < 86/88	1.000(ref)	1.000(ref)	1.000(ref)
BMI ≥ 25 and WC < 86/88	1.469 (1.412,1.528)	1.512 (1.453,1.574)	1.476 (1.418,1.536)
BMI < 25 and WC ≥ 86/88	1.215 (1.172,1.26)	1.214 (1.171,1.259)	1.230 (1.186,1.275)
BMI ≥ 25 and WC ≥ 86/88	1.662 (1.62,1.705)	1.727 (1.683,1.772)	1.729 (1.685,1.775)

*BMI* body mass index, *WC* waist circumference. Model 1: unadjusted. Model 2: adjusted for sex and age. Model 3 adjusted for Model 2 and marital status, alcohol consumption, smoking, physical activity, place of residence, cancer, stroke, psychotic illness and blood glucose

## Discussion

In this study, the prevalence of HBP increased with rising BMI and WC in the older population of Xinzheng, China, in males and females and in the entire study population. These associations remained significant after adjustment for multiple factors and restricted cubic spline analysis showed clear dose-response relationships. At the same time, this study conducted an additive interaction analysis, which concluded a significant additive interaction between BMI and WC such that the prevalence of HBP increased. To some extent, this study provides a better understanding of the association of anthropometric indicators of obesity with HBP rather than focusing on individual indices, which would be more enlightening for HBP prevention.

Our study confirmed that there was a nonlinear dose-response relationship between BMI and the risk of HBP, which was similar to previous studies [18, 19]. However, a prospective study including 1412 subjects provided evidence that an increase in BMI is associated with a linearly increased adjusted risk of developing conditions with high HBP risk [20], possibly because of estimating the relationship using categorical measures of BMI, masking the shape of the dose-response relationship. Furthermore, in our large sample (*n* = 126,123), BMI below 25 kg/m^2^ was regarded as a healthy weight for the older adults in terms of HBP prevalence. However, our proposed appropriate level for HBP were higher than those in some previous studies [21], which could be due to differences in the age range. The average age we sampled was much higher because BMI is greater in older populations, which might be a reason for the inconsistent findings, and when one of the studies stratified data by age (< 50 and ≥ 50 years), the appropriate level for the older group was <25 kg/m^2^ for men and women [22]. Contrary to the three studies above, the NHANES study suggested 27 kg/m^2^ as the value [23], but there may be innate or cultural differences between the U.S. and Asia, including dietary habits, macronutrient content, and physical activity habits. Given the marked variations in different world regions, countries and populations within countries, the use of unified range may underestimate or overestimate the health hazards [24]; thus, it is of great interest to determine the appropriate level for cardiovascular disease risks.

For WC, the relationship with HBP was reported by most studies [25, 26]. Most studies have found a positive association between WC and HBP [27], whereas a null association was found in a prospective study from European populations [26]. The low participation rate and the relatively high study drop-out rate may bias the result. We highlighted the increased risk of HBP when WC was over 88 cm and 86 cm for males and females, respectively. Our proposed WC appropriate level is higher than those reported by M Gus et al. [28], and increasing WC over time could be the cause of the difference. The worldwide upward trend in obesity has been dramatic; from 2013 to 2018, the mean WC increased from 82 cm to 86.3 cm for men and from 79.1 cm to 83.4 cm for older women [29]. However, lower appropriate level were suggested by previous studies [30, 31], and ethnic and racial differences might explain the discrepancy between different studies.

To our knowledge, the present study is the first to report a synergistic effect of higher BMI and WC on the risk of HBP in the aged. In other words, the copresence of higher BMI and WC greatly increased the risk of HBP, more than the summation of the risks due to exposure to either of them. In a cohort of 17,803 pregnant Chinese women, the copresence of a higher BMI and WC interacts to further increase the risk of gestational diabetes mellitus [32]. In addition, RISKESDAS research showed that only when general obesity or overweight coexisted with central obesity was the prevalence of HBP significantly increased [13]. This finding supported the stable relation between excess body fat and blood pressure. It is generally believed that the increased body mass would raise blood volume and cardiac output and then lead to the inadequate vasodilatation while the increased activity of the sympathetic nervous system, abnormal rennin-angiotensin-aldosterone relation and insulin resistance would arouse defects in the control of vascular resistance. These adverse vascular responses may dominate the development of obesity-associated HBP [33, 34]. Besides, natriuretic peptides and inflammatory adipokines have an active metabolic role on adipocytes, the deficit in natriuretic peptides and inflammatory adipokines may contribute to hypertension in obesity [35].

Several additional points warrant discussion. First, the findings of this cross-sectional study are not conclusive evidence of a causal relation of WC and BMI with HBP. And the measurement of blood pressure is not taking into account home or 24 h blood pressure levels. Thus, we must be cautious in interpreting the present results, and further studies are needed to clarify our findings. Second, as the study data come from the Chinese middle area among the older population, our proposed appropriate level for the indices are only valid for this population. Third, selection of the appropriate levels for BMI and WC for HBP was based on visual checking of the shapes of the OR curves. The true appropriate level of BMI and WC for HBP remained arbitrary and might deviate slightly from the selected values. However, to the best of our knowledge, our study is the first to explore the association between anthropometric Indicators of obesity and HBP among older people with a large sample size in central China, and it is of practical significance to improve relevant research. Second, this study is based on a comprehensive health check-up program, which not only contains data related to physical measurements and disease, but also includes information on demographic characteristics, physical activity, daily living habits and some blood biochemical tests, therefore, we can make full use of this information for a more comprehensive and reliable analysis.

## Conclusion

Understanding the association between BMI, WC and risks of HBP is very important because various interpretations can lead to conflicting recommendations of ideal BMIs and WCs among the older adults. The results of this study revealed that being as lean as possible within the normal range may be a best suggestion in reducing the risks of HBP. However, further cohort studies and replication studies in Chinese and other populations are needed before the results can be used in clinical practice to detect high-risk older adults for early intervention.

## Supplementary Information

**Additional file 1.**

## Data Availability

This data set is still being used for analysis. Please contact the corresponding author regarding access to the full dataset.

## Abbreviations

BMI: Body mass index
HBP: High blood pressure
WC: Waist Circumference
ANOVA: Analysis of variance
OR: Odds ratio
CI: Confidence intervals
RCS: Restricted cubic spline
CHS centers: Community health service centers
