# Supplementary material for: Association of body mass index and waist circumference with high blood pressure in older adults
Source: BMC Geriatr. 2021 Apr 19;21:260. doi: 10.1186/s12877-021-02154-5 (PMC8056549; doi:10.1186/s12877-021-02154-5)
Supplement: Supplementary file 1 — Additional file 1. [file 12877_2021_2154_MOESM1_ESM.docx]

The medical examination centers

- Xinjian Road community health service centers (CHS centers)
- Xinhua Road CHS centers
- Xinyan Road CHS centers
- Centercity CHS centers
- Xincun Town Health Center
- Xindian Town Health Center
- Guanyin Temple Town Health Center
- Lihe Town Health Center
- Hezhuang Town Health Center
- Xue Dian Town Health Center
- Meng Zhuang Town Health Center
- Guo Dian Town Health Center
- Longhu Town Health Center
- Chengguan Township Health Center
